# Tensor Approach to DOA Estimation of Coherent Signals with Electromagnetic Vector-Sensor Array

**DOI:** 10.3390/s18124320

**Published:** 2018-12-07

**Authors:** Ming-Yang Cao, Xingpeng Mao, Xiaozhuan Long, Lei Huang

**Affiliations:** 1 School of Electronics and Information Engineering, Harbin Institute of Technology, Harbin 150001, China; caomy@hit.edu.cn; 2 Key Laboratory of Marine Environmental Monitoring and Information Processing, Ministry of Industry and Information Technology, Harbin 150001, China; 3 Science and Technology on Electronic Information Control Laboratory, Chengdu 610036, China; longxiaozhuan@126.com; 4 College of Information Engineering, Shenzhen University, Shenzhen 518060, China; lhuang@szu.edu.cn

**Keywords:** direction-of-arrival estimation, electromagnetic vector-sensor array, tensor approach, coherent signals

## Abstract

This paper addresses the direction-of-arrival (DOA) estimation problem using a uniform rectangular array with electromagnetic vector-sensors in correlated/coherent signal environments. The polarization information is separated from the steering matrix to decorrelate the signals. By developing a tensorial structured received measurements of the array, we propose a tensor-based eigenvector DOA estimation method. Then we apply the forward-backward averaging to the tensor since it obeys the centro-Hermitian structure. In addition, a tensor-based polarization parameters estimation method is presented. The proposed algorithms are superior to the state-of-the-art algorithms in terms of estimation accuracy of coherent signals while only demand a modest computation burden comparing with the latter ones. Simulation results are given to demonstrate the effectiveness of the proposed methods under different scenarios.

## 1. Introduction

The electromagnetic vector-sensor array plays an important role in a variety of applications such as communication and radar [1,2,3,4,5,6]. Different from a scalar sensor, electromagnetic vector-sensor which is composed of spatially co-located three electric dipoles and three magnetic loops could be used to estimate both the direction-of-arrival (DOA) and polarization state of the impinging signals with arbitrary polarization [7,8]. The polarized vector-sensor arrays have been widely investigated for improving DOA estimation performance and resolving the polarization information of the impinging signals. In [9], an oblique, projection-based method for DOA estimation with hybrid partially and completed polarized signals are investigated. Nehorai et al. derived the stochastic Cramér-Rao bound (CRB) in [10] associated with the polarized vector-sensor array.

Many high-resolution parameter estimation approaches with polarized vector-sensor arrays such as multiple signal classification (MUSIC) or estimation of signal parameters via rotational invariance techniques (ESPRIT) are rooted in the framework of subspace decomposition and provide super-resolution ability and high DOA estimation accuracy. Yuan proposed an ESPRIT-based DOA estimation method to a polarized vector-sensor with an arbitrary degree of a polynomial-phase signal [2]. Zoltowski and Wong [5] dealt with a spatially sparse polarized vector-sensor array and could handle elevation and azimuth estimation ambiguity. A joint auto-paring two-dimensional (2D) direction-of-departure and DOA estimation approach with polarized vector-sensor MIMO radar was proposed in [6].

Note that performances of subspace-based approaches depend on an accurate estimate of the signal or noise subspace. In the real world, when encountering coherent signals, performances of subspace-based methods degrade severely because the bases of a noise subspace swap those of a signal subspace [11,12,13]. Several pre-processing techniques such as polarization smoothing algorithm (PSA) [14] and polarization difference smoothing method [15] were introduced to handle the coherent signals/multipath environments. In case of sparse polarized antenna arrays, 1D spatial smoothing was performed [16]. However, the multiway data structure (i.e., polarization, DOA and temporal dimensions) in the polarized vector-sensor array measurements is not fully utilized, which could further improve the DOA estimation performance.

The usage of multiway data, i.e., tensor, brings many opportunities to improve the DOA estimation performance [17,18]. In analogy to singular value decomposition (SVD), higher-order SVD (HOSVD) extends the concept of subspaces to multiway data, which allows subspace-based high-resolution DOA estimation methods to handle with the vector-sensor arrays. The tensor-based subspace is achieved by alternatively performing SVD to each dimension of the tensor. Furthermore, a more accurate estimate of the subspace could be obtained through performing HOSVD to the tensor when satisfying certain conditions [19]. Tensor-based parameter estimation methods [20,21] showed that the tensor-based decomposition methods were capable of providing more accurate estimates of subspaces and decorrelating correlated signals more efficiently. However, these methods require that every dimension obeys translational invariance property, which will impede their extensions to the polarized vector-sensor array since the polarization information matrix has an irregularly structure. In [22], a mode-R unfolding approach was employed to combine different unfolding modes of subspaces, leading to a better estimate of the signal subspace. Gong et al. [23] proposed a tensor-based two-fold mode-projection method which indeed is a higher-order extension of MUSIC method, and requires exhaustive searching over each parameter. The identifiability analysis, which means the maximum number of resolvable signals, was addressed through the view of CANDECOMP/PARAFAC (CP) decomposition in [24].

This paper uses a tensor-based approach with the polarized vector-sensor uniform rectangular array (URA) which could estimate both elevation and azimuth of the targets. Also, we consider a scenario where the number of samples is small comparing with the array size. Under this condition, we use direct data approach instead of the sample covariance matrix (SCM) approach. In particular, we use a preprocessing method to separate the vector-sensor polarization information matrix from the array phase factor. The proposed method reduces the singularity in the signal correlation matrix, thereby decorrelate coherent signals effectively. Next we develop a three-way tensor formulation of the received samples by exploiting the multilinear algebra, which enables us to utilize the tensorial structure in the polarized vector-sensor URA model efficiently. Then we perform forward-backward (FB) averaging of the tensor. Tensor-based ESPRIT-based algorithms associated with the proposed model are then applied to estimate both the elevation and azimuth. The proposed methods have several advantages over the state-of-art methods. (1) The proposed methods could handle the coherent signals with tensor formulation through integrating the temporal samples of signals with their polarization state information. Meanwhile, the proposed methods are able to achieve accurate estimation performances by utilizing the tensor structure inherent in the observation data. (2) Comparing with the existing two-fold method, which is also based on tensor formulation, the devised methods only require a modest computational complexity. (3) The proposed methods are more robust to the influence of errors, such as elements’ position errors and mutual coupling. Along with the polarized URA, a deterministic CRB is given as a benchmark. We give simulated performance analysis under a variety of conditions. Simulation results are provided to verify the effectiveness of the proposed methods.

This paper is organized as follows. In Section 2, we give the signal model of the polarized vector-sensor URA and steps of decorrelating the coherent signals. A tensor modeling and parameters estimation methods are proposed in Section 3. In Section 4, we give the simulations of the DOA estimation and performance analysis of the proposed methods and derive the deterministic CRB. In Section 5, numerical examples are provided to show the DOA estimation performance under coherent and correlated signals.

## 2. Signal Model

**Figure 1:**
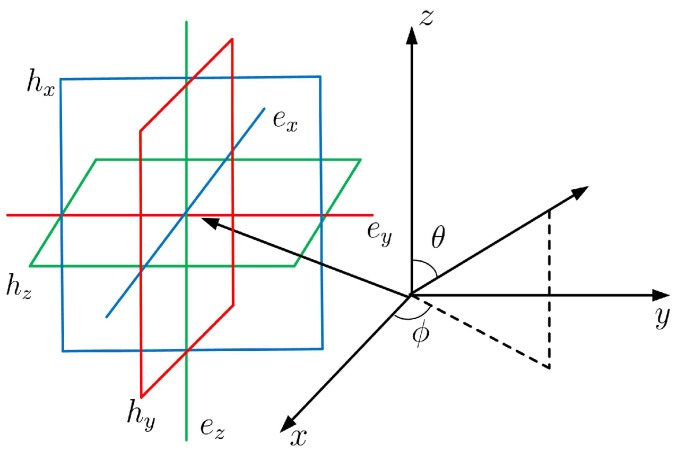
Diagram of an electromagnetic vector-sensor.

An electromagnetic vector sensor consists of six spatially co-located diversely polarized antennas, i.e., three identical and orthogonally oriented short electric dipoles, and three orthogonally small magnetic loops. The electric and magnetic field components of the *k*-th incident wavefield, which are, respectively, defined as $e_{k}\overset{▵}{=}{\left[e_{x_{k}},e_{y_{k}},e_{z_{k}}\right]}^{T}$ and $h_{k}\overset{▵}{=}{\left[h_{x_{k}},h_{y_{k}},h_{z_{k}}\right]}^{T}$, could be detected by electric dipoles and magnetic loops. The diagram of an electromagnetic vector-sensor is illustrated in Figure 1.

The ideal polarization information vector of the *k*-th signal that contains electromagnetic field information is characterized as

(1) $$p_{k}\overset{▵}{=}\left[\begin{array}{cc} \cos\phi_{k}\cos\theta_{k} & -\sin\phi_{k}\\ \sin\phi_{k}\cos\theta_{k} & \cos\phi_{k}\\ -\sin\theta_{k} & 0\\ -\sin\phi_{k} & -\cos\phi_{k}\cos\theta_{k}\\ \cos\phi_{k} & -\sin\phi_{k}\cos\theta_{k}\\ 0 & \sin\theta_{k} \end{array}\right]\left[\begin{array}{c} \sin\gamma_{k}e^{j\eta_{k}}\\ \cos\gamma_{k} \end{array}\right]$$

where $\theta_{k}\in \left[0,\pi \right],\phi_{k}\in \left[0,2\pi \right),\gamma_{k}\in \left[0,\frac{\pi }{2}\right)$, and $\eta_{k}\in \left[-\pi ,\pi \right)$ denote the *k*-th signal’s elevation measured from the positive vertical *z*-axis, azimuth, auxiliary polarization angle, polarization phase difference, respectively, ${\left(\cdot \right)}^{T}$ stands for the transpose operator.

Then the normalized Poynting vector of the *k*-th signal could be calculated as

(2) $$\begin{array}{cc} d_{k}\overset{▵}{=} & \frac{e_{k}}{\left|\right|e_{k}\left|\right|}\times \frac{h_{k}^{*}}{\left|\right|h_{k}\left|\right|}\\ \overset{▵}{=} & \left[\begin{array}{c} \mu_{k}\\ \nu_{k}\\ \omega_{k} \end{array}\right]=\left[\begin{array}{cc} \sin\theta_{k} & \cos\phi_{k}\\ \sin\theta_{k} & \sin\phi_{k}\\ \cos\theta_{k} \end{array}\right] \end{array}$$

where ${\left(\cdot \right)}^{*}$ represents the complex conjugation, ||·|| denotes the $\ell_{2}$-norm, and $\mu_{k},\nu_{k},\omega_{k}$ denotes the direction-cosine functions along the *x*-axis, *y*-axis, and *z*-axis, respectively.

**Figure 2:**
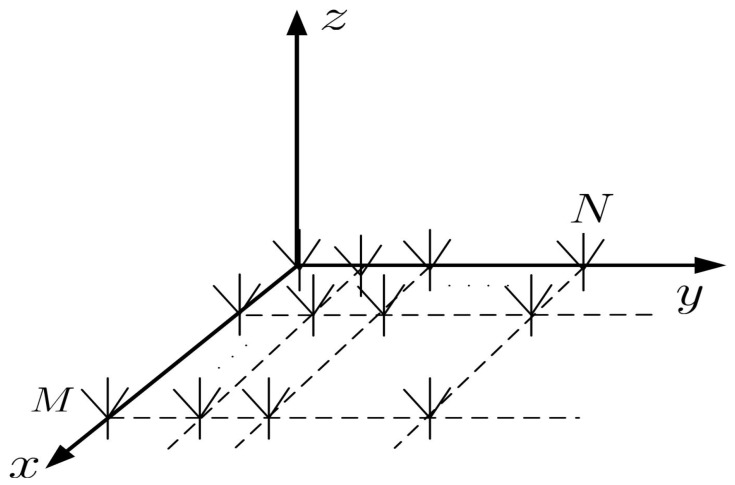
Polarized uniform rectangular array (URA) equipped with electromagnetic vector-sensors.

Consider a polarized URA consisting of M×N equally half wavelength spaced and identical six-components electromagnetic vector-sensors. This array is placed on the X-Y plane as depicted in Figure 2. Assume there are *K* far-field narrowband completed polarized signals traveled through a non-conductive homogeneous medium impinging on this array. The spatial phase factor for the (m,n)-th electromagnetic vector-sensor of the *k*-th signal is given by

(3) $$a_{mn}\left(\theta_{k},\phi_{k}\right)\overset{▵}{=}e^{j\left(m-1\right)\frac{2\pi }{\lambda }d_{x}\sin\theta_{k}\cos\phi_{k}}e^{j\left(n-1\right)\frac{2\pi }{\lambda }d_{y}\sin\theta_{k}\sin\phi_{k}}$$

where λ represents the wavelength, $d_{x}$ and $d_{y}$ stand for the adjacent vector-sensor spacing along x-axis and y-axis, respectively. Here we set $d_{x}=d_{y}=\frac{\lambda }{2}$ to avoid DOA estimation ambiguity.

Then the measurement of the (m,n)-th vector-sensor in the array at time *t* can be expressed as

(4) $$y_{mn}\left(t\right)=\sum_{k=1}^{K}s_{k}\left(t\right)\left(a_{mn}\left(\theta_{k},\phi_{k}\right)p_{k}\right)+n_{mn}\left(t\right)$$

where $y_{mn}\left(t\right)\in C^{6\times 1}$, $s_{k}\left(t\right)$ represents the *k*-th signal sampled at time *t*, $n_{mn}\left(t\right)\in C^{6\times 1}$ represents the additive complex white Gaussian noise with variance $\sigma_{n}^{2}$. Note that the output of the (m,n)-th vector-sensor is jointly decided by its electromagnetic vector-sensor $p_{k}$ and spatial phase factor $a_{mn}$. The *t*-th sample of the whole polarized vector-sensor array stacks samples of all MN vector-sensors into a vector

(5) $$\begin{array}{cc} y\left(t\right)\overset{▵}{=} & \sum_{k=1}^{K}s_{k}\left(t\right)\left(a\left(\theta_{k},\phi_{k}\right)⊗p_{k}\right)+n\left(t\right) \end{array}$$

(6) $$\begin{array}{cc} = & A_{p}s\left(t\right)+n\left(t\right) \end{array}$$

where ⊗ denotes the Kronecker product, $y\left(t\right)\in C^{6MN\times 1}$, s(t) and n(t) represent *K* signals’ waveforms and noise component at time *t*, respectively. The spatial phase factor of the M×N polarized URA associated with the *k*-th signal is defined as

(7) $$\begin{array}{cc} a\left(\theta_{k},\phi_{k}\right)\overset{▵}{=} & {\left[a_{11}\left(\theta_{k},\phi_{k}\right),\ldots ,a_{M1}\left(\theta_{k},\phi_{k}\right),\ldots ,a_{MN}\left(\theta_{k},\phi_{k}\right)\right]}^{T}\in C^{MN\times 1}\\ = & a_{y}\left(\theta_{k},\phi_{k}\right)⊗a_{x}\left(\theta_{k},\phi_{k}\right) \end{array}$$

where

(8) $$\begin{array}{cc} a_{y} & \overset{▵}{=}\left[1,\ldots ,e^{j\left(N-1\right)\pi \sin\theta_{k}\sin\phi_{k}}\right]{}^{T} \end{array}$$

(9) $$\begin{array}{cc} a_{x} & \overset{▵}{=}\left[1,\ldots ,e^{j\left(M-1\right)\pi \sin\theta_{k}\cos\phi_{k}}\right]{}^{T}. \end{array}$$

Note that $A_{p}\in C^{6MN\times K}$ represents the steering matrix

(10) $$A_{p}\overset{▵}{=}\left[a\left(\theta_{1},\phi_{1}\right)⊗p_{1},\ldots ,a\left(\theta_{K},\phi_{K}\right)⊗p_{K}\right].$$

For a total of *T* samples, we stack the received samples (6) along the temporal dimension. Thus the received matrix is given as

(11) $$Y=A_{p}S+N$$

where $Y\overset{▵}{=}\left[y\left(1\right),\ldots ,y\left(T\right)\right]$, $S\overset{▵}{=}\left[s\left(1\right),\ldots ,s\left(T\right)\right]$, and $N\overset{▵}{=}\left[n\left(1\right),\ldots ,n\left(T\right)\right]$. Throughout this paper, we use direct data approach instead of the SCM approach to estimate the DOAs. It is due to that when the number of samples is less than the size of the array, the estimate of the SCM is largely deviated from the true covariance matrix.

In order to decorrelate coherent signals, we fold the 6MN×1 received vector (6) to an MN×6 matrix as follows

(12) Z(p,q)(t)=y(6(p−1)+q)(t)

where $Z\left(t\right)\in C^{MN\times 6}$ is the new observed matrix with p=1,2,…,MN,q=1,2,⋯,6. In doing so, the polarization information are decoupled from the array phase factor as

(13) $$Z\left(t\right)=\sum_{k=1}^{K}\left(a_{y}\left(\theta_{k},\phi_{k}\right)⊗a_{x}\left(\theta_{k},\phi_{k}\right)\right)p_{k}^{T}s_{k}\left(t\right)+N_{z}\left(t\right)$$

where $N_{z}\left(t\right)$ stands for the noise item and still obeys the white Gaussian distribution. Note that Z(t) could be regarded as a sample collected by the array at time *t*. The steering vector has translational invariance property along either $a_{y}$ or $a_{x}$. Moreover, signals are decorrelated as we will see in the following part. Here we define $F\left(t\right)\overset{▵}{=}{\left[p_{1}s_{1}\left(t\right),\ldots ,p_{K}s_{K}\left(t\right)\right]}^{T}$. Thus, the corresponding signal sample covariance matrix of (13) is given as

(14) $${\hat{R}}_{F}=\frac{1}{T}\sum_{t=1}^{T}F\left(t\right)F^{H}\left(t\right)={\hat{R}}_{s}*R_{p}$$

where ${\left(\cdot \right)}^{H}$ and * stand for the conjugate transpose and Hadamard product, respectively. The signal sample covariance and polarization covariance matrices of (6) are, respectively, calculated as

(15) $$\begin{array}{cc} {\hat{R}}_{s} & =\frac{1}{T}\sum_{t=1}^{T}s\left(t\right)s{\left(t\right)}^{H} \end{array}$$

(16) $$\begin{array}{cc} R_{p} & =\left[\begin{array}{ccc} p_{1}^{T}p_{1}^{*} & \cdots & p_{1}^{T}p_{K}^{*}\\ \vdots & \ddots & \vdots \\ p_{K}^{T}p_{1}^{*} & \cdots & p_{K}^{T}p_{K}^{*} \end{array}\right]. \end{array}$$

Note that $p_{k}^{T}p_{i}^{*},k,i=1,2,\ldots ,K$ achieves the largest values if and only if k=i. Thus $R_{p}$ is a diagonal dominated matrix which will reduce singularity of the signal correlation matrix if signals are correlated. When signals are coherent, rank of the signal sample covariance matrix is equal to that of $R_{p}$. Since the polarization information of each signal differs from the other, thus we could obtain that $\mathrm{rank}({\hat{R}}_{F})=K$.

## 3. Tensor Approach

In this section, we formulate the received data (13) into a tensor. The tensorial form will reveal the array structure clearly and allow us to achieve the signal and noise subspaces more accurately. Moreover, the proposed approach admits FB averaging to further promote the DOA estimation performance.

### 3.1. Tensor Notations

**Definition** **1.** *(The n-mode tensor-matrix product): The n-mode product of a tensor $𝓐\in C^{I_{1}\times I_{2}\times \cdots \times I_{N}}$ and a matrix $D\in C^{J\times I_{n}}$ along n-th mode is given by*

(17) $$\begin{array}{c} 𝓒=𝓐\times_{n}D\\ c_{i_{1},i_{2},\ldots ,i_{n-1},j_{n},i_{n+1},\ldots ,i_{N}}=\sum_{i_{n}=1}^{I_{n}}a_{i_{1},i_{2},\ldots ,i_{N}}\cdot d_{j,i_{n}} \end{array}$$

*where $𝓒\in C^{I_{1}\times I_{2}\times \ldots \times I_{n-1}\times J_{n}\times I_{n+1}\times \ldots \times I_{N}}$.*

**Definition** **2.** *(The tensor concatenation): The concatenation of two N-way tensors $𝓐\in C^{I_{1}\times I_{2}\times \cdots \times I_{n}\times \cdots \times I_{N}}$ and $𝓓\in C^{I_{1}\times I_{2}\times \cdots \times J_{n}\times \cdots \times I_{N}}$ along n-mode is defined as $𝓑=𝓐{⊔}_{n}𝓓,$ where $𝓒\in C^{I_{1}\times I_{2}\times \ldots \times \left(I_{n}+J_{n}\right)\times \ldots \times I_{N}}$.*

**Property** **1.** *A matrix unfolding of a tensor 𝓧 along the n-mode is denoted as ${𝓧}_{\left(n\right)}$ [20]. Thus the 3-mode matricization of a three-way tensor 𝓧 can be expressed as*

(18) $${𝓧}_{\left(3\right)}=C{\left(B⊙A\right)}^{T}.$$

*where *⊙* stands for the Khatri-Rao product.*

### 3.2. Tensor Modeling

According to property 1, (13) could be regarded as a matrix form of a tensor. Thus, we fold (13) into a three-way tensor along the y-axis, x-axis of URA, and the temporal dimensions, yielding

(19) $$𝓩\left(t\right)=\sum_{k=1}^{K}a_{y}\left(\theta_{k},\phi_{k}\right)\circ a_{x}\left(\theta_{k},\phi_{k}\right)\circ \left(p_{k}s_{k}\left(t\right)\right)+𝓝\left(t\right)$$

where ∘ donates the outer product, and 𝓝(t) stands for the tensorial form of the noise item. Furthermore, (19) could be written into a more compact form which is similar to that of (13) as

(20) $$𝓩\left(t\right)=𝓐\times_{3}F\left(t\right)+𝓝\left(t\right)$$

where 𝓐 represents a three-way steering tensor. The *k*-th subtensor of 𝓐 corresponds to the steering tensor of the *k*th signal is expressed as

(21) $${𝓐}_{k}\left(\theta_{k},\phi_{k}\right)=a_{y}\left(\theta_{k},\phi_{k}\right)\circ a_{x}\left(\theta_{k},\phi_{k}\right)$$

Indeed, the subtensor of a three-way tensor represents a matrix. The proposed approach exploits the translational invariance property in the URA, while neglecting the structure of the vector sensors. For *T* samples, we define the received tensor as concatenation of each tensor at time t=1,2,…,T as

(22) $$𝓩=𝓩\left(1\right){⊔}_{3}𝓩\left(2\right){⊔}_{3}\cdots {⊔}_{3}𝓩\left(T\right).$$

### 3.3. DOA Estimation Methods

In order to perform the eigenvector-based parameter method, we give the higher-order singular value decomposition (HOSVD) of the tensor (22) as [19]

(23) $$𝓩=𝓢\times_{1}U_{1}\times_{2}U_{2}\times_{3}U_{3}.$$

where 𝓢, $U_{r},r=1,2,3$ represent the core tensor and subspaces associated with the *n*-th dimension of 𝓩. The HOSVD could be calculated by alternatively using SVD to *r*-mode matrix unfolding of 𝓩 as

(24) $${𝓩}_{\left(r\right)}=\left[\begin{array}{cc} {\hat{U}}_{r}^{\left[s\right]} & {\hat{U}}_{r}^{\left[n\right]} \end{array}\right]\left[\begin{array}{cc} {\hat{\Sigma }}_{r}^{\left[s\right]} & 0\\ 0 & {\hat{\Sigma }}_{r}^{\left[n\right]} \end{array}\right]{\left[\begin{array}{cc} {\hat{V}}_{r}^{\left[s\right]} & {\hat{V}}_{r}^{\left[n\right]} \end{array}\right]}^{H}.$$

where ${\hat{U}}_{r}^{\left[s\right]}\in C^{I_{r}\times p_{r}},p_{r}=\min\left\{I_{r},K\right\},r=1,2$, $I_{1}=M,I_{2}=N$ represent the *r*-th mode signal subspace estimate and consist singular eigenvectors associated with *K* largest singular values. The tensor-based signal subspace is then obtained by truncating the noisy observation tensor as

(25) $${\hat{𝓤}}^{\left[s\right]}={\hat{𝓢}}^{\left[s\right]}\times_{1}{\hat{U}}_{1}^{\left[s\right]}\times_{2}{\hat{U}}_{2}^{\left[s\right]}$$

and ${\hat{𝓢}}^{\left[s\right]}\in C^{p_{1}\times p_{2}\times K}$ stands for the signal core tensor. Note that the rank of signal core tensor has been restored after performing (13).

According to [19], the HOSVD provides a better signal subspace estimation than the matrix-based SVD does when K<max{M,N}. The matrix-based signal subspace ${\hat{U}}_{s}$ and tensor-based signal subspace ${\hat{𝓤}}^{\left[s\right]}$ has the following relationship

(26) $${\left[{\hat{𝓤}}^{\left[s\right]}\right]}_{\left(3\right)}^{T}=\left({\hat{T}}_{1}⊗{\hat{T}}_{2}\right){\hat{U}}_{s}$$

where ${\hat{T}}_{r}={\hat{U}}_{r}^{\left[s\right]}{\hat{U}}_{r}^{{\left[s\right]}^{H}},r=1,2$. If K<max{M,N}, ${\hat{T}}_{r}={\hat{U}}_{r}^{\left[s\right]},r=1,2$ represent projection matrices that could suppress the noise that lies outside the range of ${\hat{U}}_{r}^{\left[s\right]},r=1,2$ while maintaining the *r*-mode signal subspace. To exhibit this property, consider a matrix-based signal subspace ${\hat{U}}_{s}$ which is constructed by a Kronecker product of two sub signal subspaces, the *r*-mode signal subspace is thus defined as ${\hat{U}}_{r}^{s}\in C^{I_{r}\times p_{r}},r=1,2$. Thus we divide ${\hat{U}}_{r}^{s}$ into two parts as

(27) $${\hat{U}}_{r}^{s}={\hat{U}}_{r}^{\left[s\right]}+\Delta {\hat{U}}_{r}^{\left[s\right]}$$

where $\Delta {\hat{U}}_{r}^{\left[s\right]}$ lies outside the range of $U_{r}^{\left[s\right]}$. Performing the projection matrix to ${\hat{U}}_{r}^{s}$ as in (46), we have

(28) $${\hat{T}}_{r}{\hat{U}}_{r}^{s}={\hat{U}}_{r}^{\left[s\right]}{\hat{U}}_{r}^{{\left[s\right]}^{H}}\left({\hat{U}}_{r}^{\left[s\right]}+\Delta {\hat{U}}_{r}^{\left[s\right]}\right)={\hat{U}}_{r}^{\left[s\right]}.$$

Therefore, the signal subspace ${\hat{U}}_{s}$ is filtered with respect to the *r*-mode signal subspaces obtained through (24). When K≥max{M,N}, ${\hat{T}}_{r}$ becomes identity matrix, the tensor-based signal subspace is as the same as the matrix-based signal subspace.

Similar to the matrix cases, the relationship between the steering tensor and corresponding signal subspace is

(29) $$𝓐\approx {\hat{𝓤}}^{\left[s\right]}\times_{3}T$$

where T denotes a nonsingular transform matrix. The rotational invariance property holds for the tensor-based signal subspace, and it can be exploited for estimating both elevation and azimuth. Thus we have

(30) $$\begin{array}{c} {\hat{𝓤}}^{\left[s\right]}\times_{1}J_{1}^{\left(1\right)}\times_{3}\Theta \;\approx \;{\hat{𝓤}}^{\left[s\right]}\times_{1}J_{2}^{\left(1\right)} \end{array}$$

(31) $$\begin{array}{c} {\hat{𝓤}}^{\left[s\right]}\times_{2}J_{1}^{\left(2\right)}\times_{3}\Phi \;\approx \;{\hat{𝓤}}^{\left[s\right]}\times_{2}J_{2}^{\left(2\right)} \end{array}$$

where Θ and Φ are the diagonal matrices carrying the direction cosine functions, that is,

(32) $$\begin{array}{c} \Theta \overset{▵}{=}\mathrm{diag}\left\{\left[e^{-j\pi \sin\theta_{1}\cos\phi_{1}},\ldots ,e^{-j\pi \sin\theta_{K}\cos\phi_{K}}\right]\right\} \end{array}$$

(33) $$\begin{array}{c} \Phi \overset{▵}{=}\mathrm{diag}\left\{\left[e^{-j\pi \sin\theta_{1}\sin\phi_{1}},\ldots ,e^{-j\pi \sin\theta_{K}\sin\phi_{K}}\right]\right\} \end{array}$$

and $J_{1}^{\left(r\right)}$ as well as $J_{2}^{\left(r\right)}$ stand for the selection matrices of each dimension for r=1,2, which are defined as

(34) $$\begin{array}{c} J_{1}^{\left(r\right)}\overset{▵}{=}\left[I_{I_{r}-1}\;0_{(I_{r}-1)\times 1}\right] \end{array}$$

(35) $$\begin{array}{c} J_{2}^{\left(r\right)}\overset{▵}{=}\left[0_{(I_{r}-1)\times 1}\;I_{I_{r}-1}\right] \end{array}$$

where $I_{I_{r}},r=1,2$ stands for the identity matrix with size $I_{r}$. In (30) and (31), the translational invariance property holds in the left- and right-hand sides of the expressions. However, the estimation performance maybe not good since ${\hat{𝓤}}^{\left[s\right]}$ has a large size.

In accordance with the TEV method [20], we separate the tensor-based signal subspace into several small parts along the signal dimension to further reveal the link between the steering tensor and signal subspace, that is

(36) $${\hat{𝓤}}^{\left[s\right]}={𝓠}_{1}{⊔}_{3}{𝓠}_{2}{⊔}_{3}\cdots {⊔}_{3}{𝓠}_{K}.$$

For the *k*-th signal, we have

(37) $${𝓐}_{k}={𝓠}_{k}\times G_{k},\;k=1,2,\ldots ,K$$

where $G_{k},k=1,2$ are non-singular matrices. Recalling the relationship in (21), we find out that ${𝓠}_{k}$ represents the signal subspace associated with the *k*-th signal dimension. Applying SVD to ${𝓠}_{k}$ yields

(38) $$\begin{array}{c} {𝓠}_{k}=\xi_{k}q_{k,1}\circ q_{k,2}. \end{array}$$

It can be observed that $q_{k,r}$ spans the same subspace as the *r*-th dimension of ${𝓐}_{k}$. Using the linear prediction property [25] and the manipulations in the ESPRIT method, we obtain the equalities to determine the elevation and azimuth as

(39) $$\begin{array}{c} J_{1}^{\left(1\right)}q_{k,1}=e^{-j\pi \sin\theta_{k}\cos\phi_{k}}J_{2}^{\left(1\right)}q_{k,1} \end{array}$$

(40) $$\begin{array}{c} J_{1}^{\left(1\right)}q_{k,2}=e^{-j\pi \sin\theta_{k}\sin\phi_{k}}J_{2}^{\left(1\right)}q_{k,2}. \end{array}$$

This method operates on each dimension of the signal subspace separately. We could obtain the elevation and azimuth estimates by solving (39) and (40) through least squares (LS) or structured least squares (SLS) algorithms. Note that the elevation and azimuth are auto-paring since the *K*-th subtensor in (38) associated with *k*-th signal.

In addition, the FB averaging could be performed to the tensor model. However, the FB averaging could not be directly applied to Y since its steering matrix does not obey a Vandermonde structure. Instead, we perform the FB averaging to 𝓩, thus

(41) $${𝓩}_{\mathrm{FB}}=𝓩{⊔}_{3}\left({𝓩}^{*}\times_{1}M_{I_{1}}\times_{2}M_{I_{2}}\right)$$

where $M_{I_{r}},r=1,2$ represents a $I_{r}\times I_{r}$ matrix with ones on its anti-diagonal and zeroes elsewhere. After obtaining ${𝓩}_{\mathrm{FB}}$, the rest are the same as the steps we take in the proposed VS-TEV method.

The proposed VS-TEV and FB-VS-TEV methods are tabulated in Table 1.

### 3.4. Polarization Parameters Estimation

In this paper, we mainly focus on the DOA estimation problem with polarized vector-sensor URA. However, the vector-sensors carry the polarization information of the signals, i.e., auxiliary polarization angle and polarization phase difference. Herein we briefly derive a tensor-based polarization information estimation method as a complementary part to the proposed tensor approach. The array measurement at time *t* is reformulated as

(42) X(p,q)(t)=y(p+6(q−1))(t)

where $X\left(t\right)\in C^{6M\times N}$ and

(43) $$X\left(t\right)=\sum_{k=1}^{K}\left(a_{y}\left(\theta_{k},\phi_{k}\right)⊗p_{k}\right)a_{x}^{T}\left(\theta_{k},\phi_{k}\right)s_{k}\left(t\right)+N_{p}\left(t\right)$$

where $N_{p}\left(t\right)\in C^{6M\times N}$ stands for the complex white Gaussian noise. Similar as in (19)–(22), we construct a tensorial form of *T* samples as

(44) $$𝓧={𝓐}_{p}\times_{3}{𝓕}_{p}+{𝓝}_{p}$$

where ${𝓐}_{p}\in C^{6\times M\times K}$ represents the steering tensor, ${𝓕}_{p}$ and ${𝓝}_{p}$ stands for the signal and noise components, respectively. Note that the signals are noncoherent. The *k*-th subtensor of ${𝓐}_{p}$ has the following property

(45) $${𝓐}_{pk}=p_{k}\left(\theta_{k},\phi_{k},\eta_{k},\gamma_{k}\right)\circ a_{y}\left(\theta_{k},\phi_{k}\right).$$

Note that ${𝓐}_{pk}$ has a translational invariance property along $a_{y}$. Performing HOSVD to 𝓧, the tensor-based signal subspace is obtained as

(46) $${\hat{𝓤}}_{p}^{\left[s\right]}={\hat{𝓢}}_{p}^{\left[s\right]}\times_{1}{\hat{U}}_{p,1}^{\left[s\right]}\times_{2}{\hat{U}}_{p,2}^{\left[s\right]}.$$

where ${\hat{𝓢}}_{p}^{\left[s\right]}$, ${\hat{U}}_{p,i}^{\left[s\right]},i=1,2$ stand for the core tensor and the *i*-mode signal subspace, respectively, and the dimensions of them are decided as the same way as in (25). There exists a nonsingular matrix $G\in C^{K\times K}$ that

(47) $${\hat{𝓤}}_{p}^{\left[s\right]}={𝓐}_{p}\times_{3}G.$$

Here we develop the ESPRIT-lie method to estimate both η and γ. Consider two subtensors of ${\hat{𝓤}}_{p}^{\left[s\right]}$, and they have the following

(48) $$\begin{array}{cc} {\hat{U}}_{p1} & ={\hat{𝓤}}_{p}^{\left[s\right]}\times_{1}j^{\left(1\right)} \end{array}$$

(49) $$\begin{array}{cc} {\hat{U}}_{p2} & ={\hat{𝓤}}_{p}^{\left[s\right]}\times_{1}j^{\left(2\right)} \end{array}$$

where ${\hat{U}}_{pi}\in C^{6\times K},i=1,2$ and the selection vectors are defined as

(50) $$\begin{array}{c} j^{\left(1\right)}\overset{▵}{=}\left[1\;0_{1\times (N-1)}\right] \end{array}$$

(51) $$\begin{array}{c} j^{\left(2\right)}\overset{▵}{=}\left[0\;1\;0_{1\times (N-2)}\right]. \end{array}$$

Consider a noiseless case, thus we take advantage of the rational invariance property in ${\hat{𝓤}}_{p}^{\left[s\right]}$

(52) $$\begin{array}{cc} {\hat{U}}_{p1}\; & =\;PG \end{array}$$

(53) $$\begin{array}{cc} {\hat{U}}_{p2}\; & =\;P\Phi G \end{array}$$

where ${\hat{U}}_{pi}\in C^{6\times K},i=1,2$. There exists a unique nonsingular matrix Ψ satisfies that

(54) $${\hat{U}}_{p1}\Psi ={\hat{U}}_{p2}$$

Note that Ψ could be estimated through LS, and its eigenvalue decomposition have the following expression

(55) $$\Psi ={\left({\hat{U}}_{p1}^{H}{\hat{U}}_{p1}\right)}^{-1}{\hat{U}}_{p1}^{H}{\hat{U}}_{p2}=G^{-1}\Phi G.$$

Here G stands for the right eigenvectors of Ψ. Thus we obtain the estimate of P as

(56) $$\hat{P}={\hat{U}}_{p1}G^{-1}.$$

Since we have already obtained an estimate of $\hat{\theta }$ and $\hat{\phi }$. Substituting $\hat{\theta }$ and $\hat{\phi }$, and then we achieve an estimate the polarization information. The polarization parameters, i.e., η,γ, associated with *k*-th signal are hence calculated by utilizing the structure of polarization information vector as in Ref. [4]. Note that the DOA and polarization parameters are auto-pairing since we use eigenvectors to estimate the polarization parameters instead of eigenvalues.

## 4. Performance Analysis

In this section, we firstly derive the expression of deterministic CRB of this model. Subsequently, we consider the influences of different kinds of errors, i.e., array elements’ position errors and mutual couplings within electromagnetic vector-sensors, on the proposed methods [26,27]. Finally, we analyze the estimation performance of devised approaches under various signal-to-noise ratio (SNR) and the numbers of samples.

### 4.1. Derivation of CRB

In this part, we derive the deterministic CRB of the DOA with the polarized vector-sensor URA. Since the number of samples is much less than the dimension of the array, and hence we cannot obtain an accurate estimate of the sample covariance matrix. Therefore, this is more reasonable that we use deterministic CRB to verify the effectiveness of the proposed methods that are based on direct data approach. The deterministic CRB of the multidimensional parameter estimation has been derived in Ref. [28]. Furthermore, the polarized URA could be regarded as a multidimensional model. Among all the parameters, elevation and azimuth are particularly considered and are

(57) $$\mu \overset{▵}{=}{\left[\theta^{T},\phi^{T}\right]}^{T}$$

and the CRB for accuracy of the parameter vector (57) estimate can therefore be written in our notations as

(58) CRB(μ)=σn22diag∑t=1TReStHDH×(I−Ap(ApHAp)−1ApH)DSt−1

where

(59) $$\begin{array}{cc} S_{t}\overset{▵}{=} & I_{2}⊗\mathrm{diag}\left(s\left(t\right)\right) \end{array}$$

(60) $$\begin{array}{cc} D\overset{▵}{=} & \left[D_{\theta },D_{\phi }\right] \end{array}$$

(61) $$\begin{array}{cc} D_{\theta }\overset{▵}{=} & \left(\left(A_{y\theta }⊙A_{x}\right)\right)⊙P+\left(\left(A_{y}⊙A_{x\theta }\right)\right)⊙P+\left(\left(A_{y}⊙A_{x}\right)\right)⊙P_{\theta } \end{array}$$

(62) $$\begin{array}{cc} D_{\phi }\overset{▵}{=} & \left(\left(A_{y\phi }⊙A_{x}\right)\right)⊙P+\left(\left(A_{y}⊙A_{x\phi }\right)\right)⊙P+\left(\left(A_{y}⊙A_{x}\right)\right)⊙P_{\phi } \end{array}$$

with $A_{y\theta },A_{x\theta },P_{\theta },A_{y\phi },A_{x\phi },P_{\phi }$ being the first order derivation of $A_{y},A_{x},P$, which are the steering matrix of *K* signals of $a_{y},a_{x},p$, with respect to elevation and azimuth, respectively.

### 4.2. Array Elements’ Position Errors

The proposed methods require the translational invariance property along both $a_{y}$ and $a_{x}$. When there exists elements’ position errors of each vector-sensor, the $a_{y}$ and $a_{x}$ no longer strictly obey the translational invariance property, which will degrade the DOA estimation performance. We analysis the robustness of the proposed methods against the elements’ position errors. The position errors of the (m,n)-th vector-sensor in the URA are defined as $\left(\Delta d_{x_{m}},\Delta d_{y_{n}}\right)$ Note that both $\Delta d_{x_{m}}$ and $\Delta d_{y_{n}}$ have no influences on the polarization information vectors, thus we define the spatial factor perturbed by array elements’ position errors as

(63) $${\tilde{a}}_{mn}\left(\theta_{k},\phi_{k}\right)\overset{▵}{=}e^{j\frac{2\pi }{\lambda }\left(\left(m-1\right)d_{x}+\Delta d_{x_{m}}\right)\sin\theta_{k}\cos\phi_{k}}e^{j\frac{2\pi }{\lambda }\left(\left(n-1\right)d_{y}+\Delta d_{y_{n}}\right)\sin\theta_{k}\sin\phi_{k}}.$$

Thus, the *t*-th sample observed by the (m,n)-th vector-sensor is expressed as

(64) $$y_{mn}\left(t\right)=\sum_{k=1}^{K}s_{k}\left(t\right)\left({\tilde{a}}_{mn}\left(\theta_{k},\phi_{k}\right)p_{k}\right)+n_{mn}\left(t\right).$$

It is obvious that the position errors will breach translational invariance property of the steering matrix of polarized URA. Thus we analysis the impact of elements’ position errors on the DOA estimation performance of two proposed methods. The DOAs of two coherent signals are set as $\theta =[30^{\circ },45^{\circ }]$, $\phi =[15^{\circ },30^{\circ }]$ and $\gamma =\left[20^{\circ },40^{\circ }\right],\eta =\left[30^{\circ },60^{\circ }\right]$. Throughout all the simulations, we assume that the frequencies of the signals are fixed at 10 MHz, and the adjacent vector-sensor spacing is set as half-wavelength. The number of samples is fixed at T=20, and the size of URA is 8×7. In practice, the errors always behave randomly. For simplicity, we assume the random position errors $\Delta d_{x_{m}},m=1,\ldots ,M$ and $\Delta d_{y_{n}},n=1,\ldots ,N$ obey stochastic Gaussian processes $N(0,\Delta^{2})$. The root mean square error (RMSE) of a parameter is defined respectively as

(65) $${\mathrm{RMSE}}_{\lambda }=\sqrt{E\left\{\frac{1}{K}\sum_{k=1}^{K}{\left({\hat{\lambda }}_{k}-\lambda_{k}\right)}^{2}\right\}}.$$

where E{.} stand for the expectation operator, and λ could represent θ,ϕ,η,γ, respectively. In addition, we define the SNR as $10\log_{10}(∥A_{p}{S∥}_{F}^{2}/{∥N∥}_{F}^{2})$.

**Figure 3:**
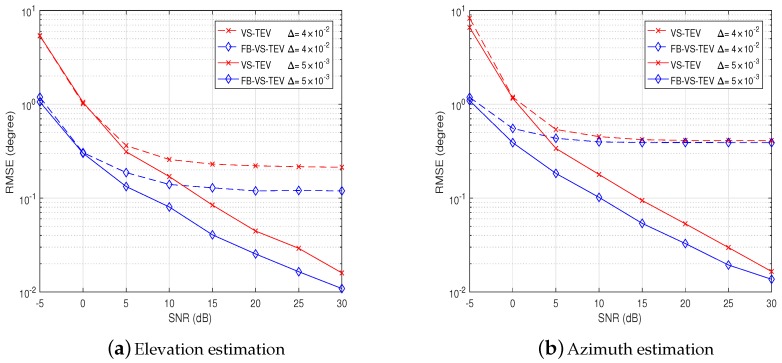
Performance analysis of elements’ position errors: Root mean square error (RMSE) versus signal-to-noise ratio (SNR) for two coherent signals with *T* = 20.

In Figure 3a, the elevation estimation performances of the proposed VS-TEV and FB-VS-TEV methods become better when SNR increases if we set $\Delta =5\times 10^{-3}$. Moreover, the FB-VS-TEV method performs better than the VS-TEV method. However, the elevation estimation performances degrade when the perturbation error becomes larger at the same SNR, e.g., $\Delta =4\times 10^{-2}$. In this case, it can be observed that both the proposed methods perform much worse when SNR is sufficiently large, say, SNR > 5 dB, and the elevation estimation performances converge to non-zero constants when SNR > 15 dB. This is due to the fact that the performance degradation mainly caused by the position errors rather than noise when SNR is high. The same phenomenon happens in the azimuth estimation performance as shown in Figure 3b. If we set $\Delta =4\times 10^{-2}$, it is observed that the position errors have little effect on the azimuth estimation performance when SNR < 5 dB. When SNR > 10 dB, the RMSEs of azimuth estimation do not decrease as the SNR increases. Again, the azimuth estimation performance behaves better as the SNR increases if we set the mutual coupling coefficient as a smaller number, say, $\Delta =5\times 10^{-3}$. Moreover, the FB-VS-TEV method is more robust against the elements position errors compared with the VS-TEV method shown in Figure 3.

### 4.3. Mutual Coupling Effect

Since a vector-sensor consists of co-located electric dipoles and magnetic loops, the mutual couplings between them cannot be neglected. In this subsection, we study the performance analysis of the proposed methods when the mutual coupling exists within each vector-sensors. Note that the adjacent vector-sensor spacing is set as half wavelength, which is fifteen meters if frequencies of signals are set as 10 MHz. Thus, the mutual couplings between different vector-sensors are very small. This is why we only consider the mutual couplings within the electromagnetic vector-sensors. Based on the physical structure of the electromagnetic vector-sensor, the symmetric mutual coupling matrix of the (m,n)-th vector-sensor is defined as Ref. [29]

(66) $$C_{mn}=\left[\begin{array}{cccccc} 1 & c_{mn}^{1} & c_{mn}^{1} & c_{mn}^{2} & c_{mn}^{3} & c_{mn}^{3}\\ c_{mn}^{1} & 1 & c_{mn}^{1} & c_{mn}^{3} & c_{mn}^{2} & c_{mn}^{3}\\ c_{mn}^{1} & c_{mn}^{1} & 1 & c_{mn}^{3} & c_{mn}^{3} & c_{mn}^{2}\\ c_{mn}^{2} & c_{mn}^{3} & c_{mn}^{3} & 1 & c_{mn}^{1} & c_{mn}^{1}\\ c_{mn}^{3} & c_{mn}^{2} & c_{mn}^{3} & c_{mn}^{1} & 1 & c_{mn}^{1}\\ c_{mn}^{3} & c_{mn}^{3} & c_{mn}^{2} & c_{mn}^{1} & c_{mn}^{1} & 1 \end{array}\right].$$

where $c_{mn}^{1},c_{mn}^{2}$, and $c_{mn}^{3}$ represent the mutual coupling coefficients between different electric dipoles and different magnetic loops, respectively. Thus the *t*-th sample of the (m,n)-th electromagnetic vector-sensor with mutual coupling effect is

(67) $$y_{mn}\left(t\right)=\sum_{k=1}^{K}s_{k}\left(t\right)\left(a_{mn}\left(\theta_{k},\phi_{k}\right)C_{mn}p_{k}\right)+n_{mn}\left(t\right).$$

Note that the mutual coupling will affect the steering matrix of the vector-sensor array, which will in turn influence the DOA estimation performance. Here we give different mutual coupling levels to examine the robustness of the proposed VS-TEV and FB-VS-TEV methods against mutual coupling effect. To emphasis the influence of mutual coupling, we suppose that the polarized URA does not have elements’ position errors. In simulations, we define the mutual coupling coefficients of the (m,n)-th vector-sensor as $c_{mn}^{i},i=1,2,3$ which obey stochastic Gaussian processes $N(0,\delta^{2})$. The other settings are the same as those in Figure 3.

**Figure 4:**
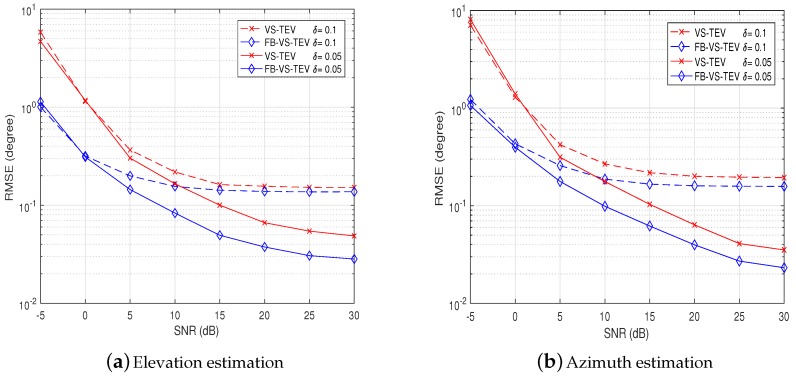
Performance analysis of proposed methods with mutual coupling: RMSE versus SNR for two coherent signals with *T* = 20.

It can be observed that the RMSEs of the proposed methods do not increase for δ=0.1 when SNR is larger than 15 dB. This in turn indicates that the errors caused by mutual coupling are much larger than noise. If we set δ=0.05, the influences of mutual coupling are very small, and the noise influence becomes dominated, as can be seen in Figure 4. On the other hand, it is observed in Figure 4 that the FB-VS-TEV method behaves better than the VS-TEV method.

### 4.4. Performance Analysis of the Proposed Methods

In this subsection, we give the performance analysis of the proposed DOA and polarization estimation methods based on simulations. We vary several parameters such as SNR, the number of samples, correlation coefficients which will influence the DOA and polarization estimation performances to testify the effectiveness of the proposed methods under a variety of cases. Throughout all the simulations, the size of URA is set as 8×7.

**Figure 5:**
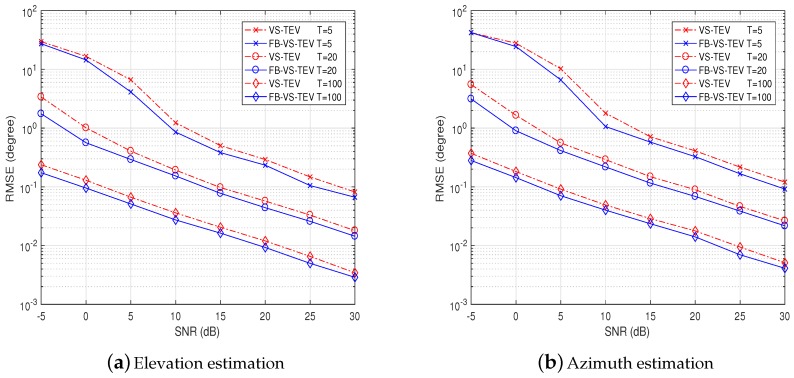
RMSE performance of proposed methods versus SNR of two coherent signals with *T* = [5, 20, 100].

In Figure 5, we study the elevation and azimuth estimation performance versus SNR with different numbers of samples. The parameters of two coherent signals are set as $\theta =[25^{\circ },35^{\circ }]$, $\phi =[15^{\circ },25^{\circ }]$ and $\gamma =\left[20^{\circ },40^{\circ }\right],\eta =\left[30^{\circ },60^{\circ }\right]$. It is observed that the DOA RMSEs of the proposed methods decrease when the SNR increases. As the number of samples increases, the proposed methods have lower DOA RMSEs at the same SNRs. Also, the FB-VS-TEV method outperform the VS-TEV method.

**Figure 6:**
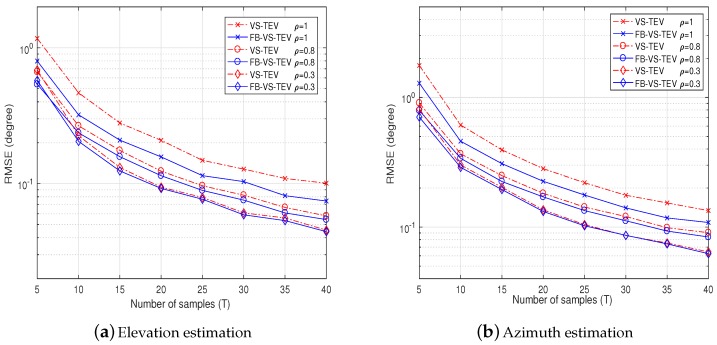
Direction-of-arrival (DOA) RMSE performance of proposed methods versus the number of samples for two correlated signals with *ρ* = 0.3, 0.8, 1, respectively. The SNR is fixed at 10 dB.

In Figure 6, we investigate the elevation and azimuth estimation performance versus the number of samples with different correlation coefficients. The correlation coefficient of two signals with zero mean is given as

(68) $$\rho =\frac{E\{s_{1}\left(t\right)s_{2}\left(t\right)\}}{\sqrt{E\left\{s_{1}^{2}\left(t\right)\right\}E\left\{s_{2}^{2}\left(t\right)\right\}}}.$$

The settings of DOAs and polarization information is as the same as in Figure 5, and SNR is set as 10 dB. As we can see, the DOAs RMSE decreases when the number of samples increases. Also, when the correlation coefficients are high, the DOA RMSE performances become worse. The proposed VS-TEV and FB-VS-TEV methods have almost the same performance when the correlation coefficient is small.

**Figure 7:**
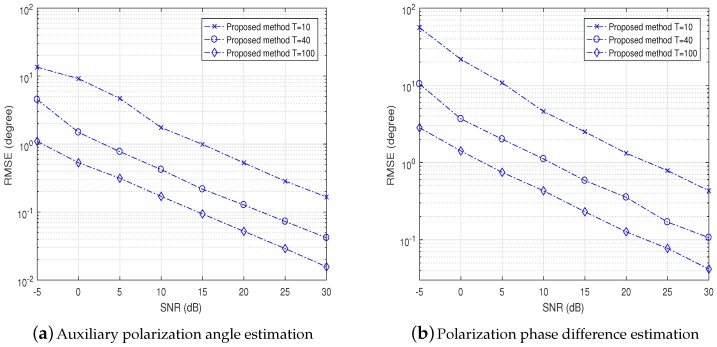
Auxiliary polarization angle and polarization phase difference estimation RMSE performance of proposed methods versus SNR for two coherent signals with *T* = 10, 40, 100, respectively.

In Figure 7a,b, we vary SNR and the number of samples to show the auxiliary polarization angle and polarization phase difference estimation performances of two coherent signals, respectively. We observe that the proposed method could estimate η and γ accurately when the SNR or the number of samples is small.

### 4.5. Computational Complexity Analysis

The computational complexity of the propose method is given as follows. The HOSVD of measurement tensor Z demands O(24TMNK), truncated HOSVD of *K* subtensors demands O(6MNK) and SLS update demands $O(K\sum_{r=1}^{3}M_{r}^{3})$. For the spectral searching technique, e.g., two-fold mode projection DOA estimation method, the computational complexity is $O\left(6NM^{2}K\right)+O\left(36N^{2}MK\right)$+$O(12M^{2}NJ+72MN^{2}J+24MNJ)$, where *J* stands for the number of grid points of the searing region, and usually we have J≫N,J≫M. Comparing with the spectral searching methods, the proposed methods require less computational burden. The average CPU time of two-fold projection, PSA, and the proposed VS-TEV, FB-VS-TEV methods are listed below. We set the size of URA as 8×7, T=10, and K=2. For two-fold projection method, if we set the searching grid as $0.01^{\circ }$, thus J=18,000 for a 1-D searching region of size $\left[-90^{\circ }\;90^{\circ }\right]$. In practice, we could apply refined technique to decrease *J*. The average CPU time of all the methods is based on a PC equipped with a Core i3 3.7 GHz processor, 8GB RAM and Matlab R2016b version. Computation time of four methods are listed in Table 2.

Based on Table 2, the proposed VS-TEV and FB-VS-TEV methods demand much less computational burdens than those of two-fold methods. It is because the proposed methods are based on polynomial rooting instead of exhaustive grid searching. However, the proposed methods involve HOSVD that needs more computations than the matrix-based SVD. Thus, the VS-TEV and FB-VS-TEV methods consume more computational time than the PSA method.

## 5. Simulations

In this part, we discuss two cases that two signals are coherent and noncoherent i.e., the correlation coefficient of two signals varies from 1 to 0. For the purpose of comparisons, the results of the approaches, i.e., PSA [14] and two-fold projection [23] are included in the first case. Since TEV method cannot resolve two coherent signals, we do not include it in the case of coherent signals. Since two-fold projection method demands an exhaustive searching, we set the searching area as a small spatial region which includes the DOAs. Also, we give the deterministic polarized vector-sensor URA’s CRB as a benchmark to examine the effectiveness of the proposed method. For all simulations, the Monte-Carlo trials for each experiment is set as 1000. Here we define total RMSE (TRMSE) of elevation and azimuth as

(69) $$\mathrm{TRMSE}=\sqrt{E\left\{\frac{1}{K}\sum_{k=1}^{K}{\left({\hat{\theta }}_{k}-\theta_{k}\right)}^{2}+{\left({\hat{\phi }}_{k}-\phi_{k}\right)}^{2}\right\}}.$$

### 5.1. The Case of Coherent Signals

First, we provide the simulation results for the elevation and azimuth root mean square error (RMSE) performances of the proposed VS-TEV, FB-VS-TEV. Throughout all the simulations, the size of URA is set as 6×6.

**Figure 8:**
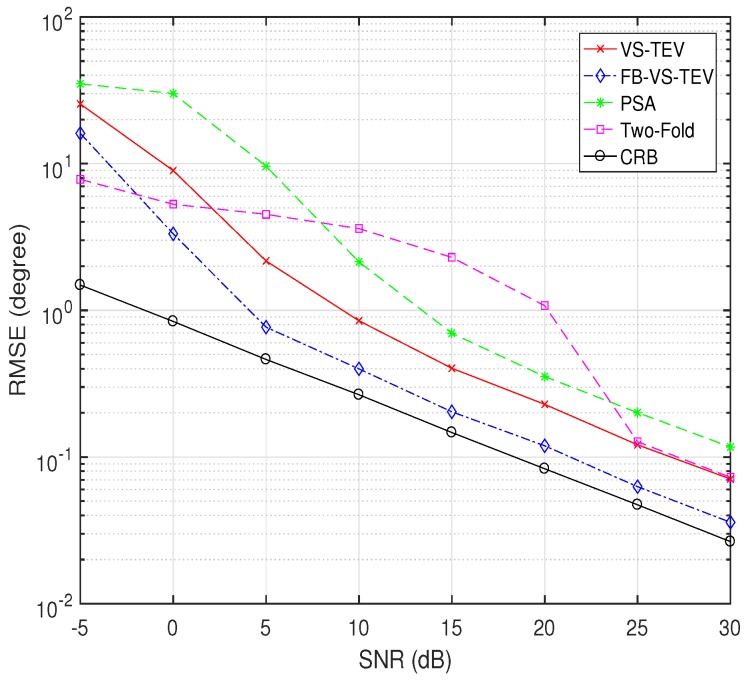
RMSE of elevation and azimuth versus SNR for two coherent signals with T=10.

In Figure 8, we compare performances of all methods while varying SNR. The number of snapshots is fixed to 10. Two signals are assumed to impinge on the array with $\theta =[30^{\circ },45^{\circ }]$, $\phi =[15^{\circ },30^{\circ }]$ and $\gamma =\left[20^{\circ },40^{\circ }\right],\eta =\left[30^{\circ },60^{\circ }\right]$. It is observed that the performances of all algorithms improve when SNR increases. And two-fold projection methods suffer from a more severe threshold effect when SNR is small. The proposed FB-VS-TEV outperforms all the other algorithms when SNR > −5 dB. The VS-TEV and two-fold projection method has almost the same RMSE performance when SNR > 25 dB, however the latter method requires more computations. The RMSE performance of the FB-VS-TEV method is close to CRB when SNR ≥ 5 dB.

**Figure 9:**
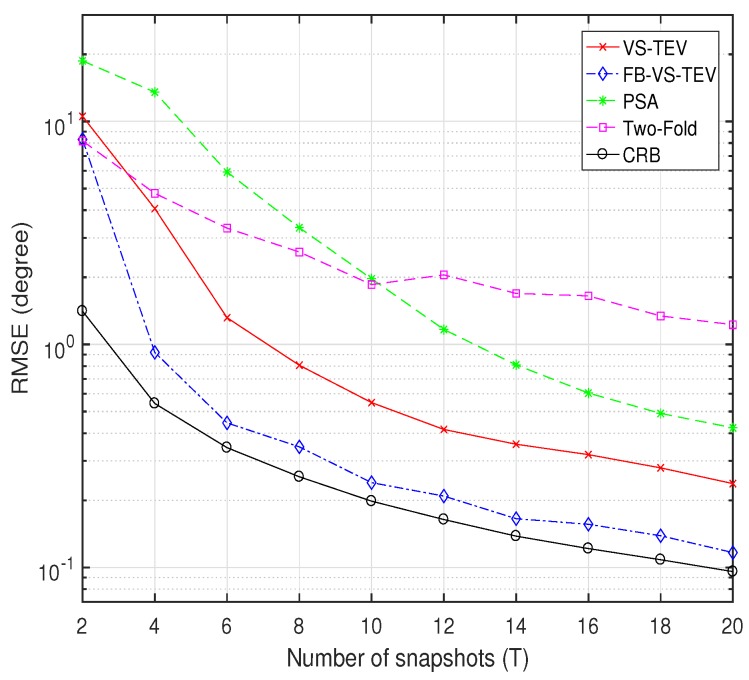
RMSE of elevation and azimuth versus the number of samples for two coherent signals with SNR = 10 dB.

In Figure 9, we investigate the RMSE performance versus the number of samples with SNR=10dB. The DOA and polarization information settings are as the same as that in Figure 8. It can be observed that in Figure 9, the FB-VS-TEV method outperform all the other methods and is close to CRB when T≥3. The proposed VS-TEV method has more accurate estimates than PSA and two-fold methods when T≥4.

**Figure 10:**
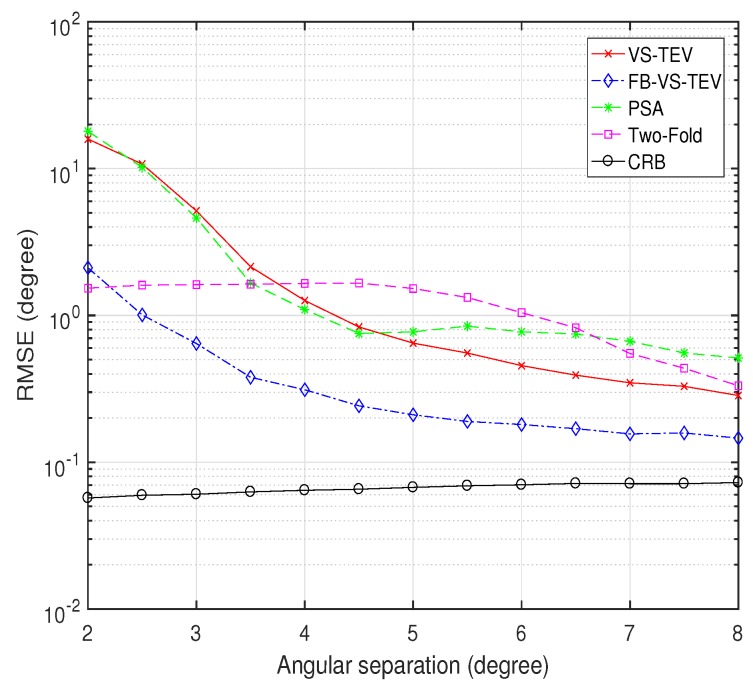
RMSE of elevation and azimuth versus angular separation for two coherent signals with T=10, SNR = 10 dB.

In another example, Figure 10 shows the RMSE performances of all these methods versus the angular separation. We fix DOA of the first signal at $\left(\theta_{1},\phi_{1}\right)=\left[30^{\circ },15^{\circ }\right]$ and vary DOA of the second target to examine RMSE performances of all the methods. The polarization parameters of two signals are set as $\gamma =\left[20^{\circ },40^{\circ }\right],\eta =\left[30^{\circ },60^{\circ }\right]$. SNR is set as 20 dB, and the number of samples is fixed at T=10. As we can see, the FB-VS-TEV method has the lowest DOA estimation RMSE among all the methods when angular separation is larger than $2.5^{\circ }$. When angular separation is larger than $4^{\circ }$, VS-TEV method performs better than the other method except the FB-VS-TEV method. In order to lower the computational burden of the two-fold projection method we set a small size spatial searching region, for which the RMSE of this method goes flat when angular separation is smaller than $5^{\circ }$. Note that the CRB does not decrease as the angular separation increases. It is because that the CRB is not only a function of DOA, but is also influenced by the polarization information.

**Figure 11:**
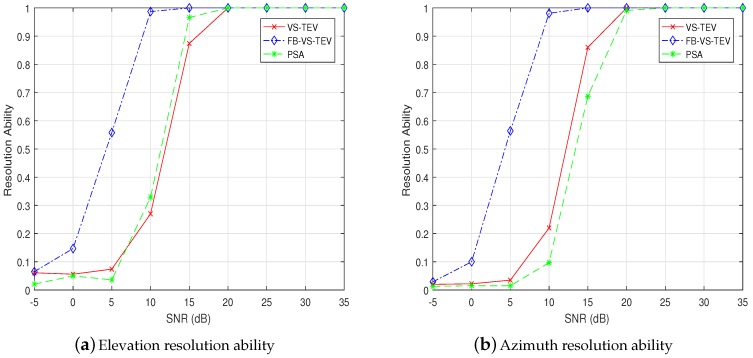
Resolution ability of elevation and azimuth versus SNR for two coherent signals with *T* = 20.

Thirdly, we observe the resolution abilities of all the mentioned methods. The signals are defined as resolvable if the following condition is satisfied for the elevation estimation [30]

(70) $$|{\hat{\theta }}_{k}-\theta_{k}|<\frac{|\theta_{1}-\theta_{2}|}{2}$$

where ${\hat{\theta }}_{k},k=1,2$ represent the estimated elevation parameters of two signals. This above criterion is also suitable for testing the azimuth resolution ability if we replace θ with ϕ in Equation (70). In this simulation, the DOAs of two coherent signals are set as $\theta =[25^{\circ },29^{\circ }]$, $\phi =[15^{\circ },19^{\circ }]$ and $\gamma =[20^{\circ },40^{\circ }],$ $\eta =[30^{\circ },60^{\circ }]$, and the number of snapshots is fixed as 20. In Figure 11, it can be seen that all the methods have poor elevation and azimuth resolution abilities when SNR < 5 dB. When SNR > 10 dB, the success ratio of resolution of the FB-VS-TEV method converges to one. The VS-TEV and PSA methods have almost the same resolution abilities and converge to one when SNR > 15 dB. Note that the two-fold method has a rather poor resolution ability that we do not include it for comparison in this simulation.

### 5.2. The Case of Noncoherent Signals

**Figure 12:**
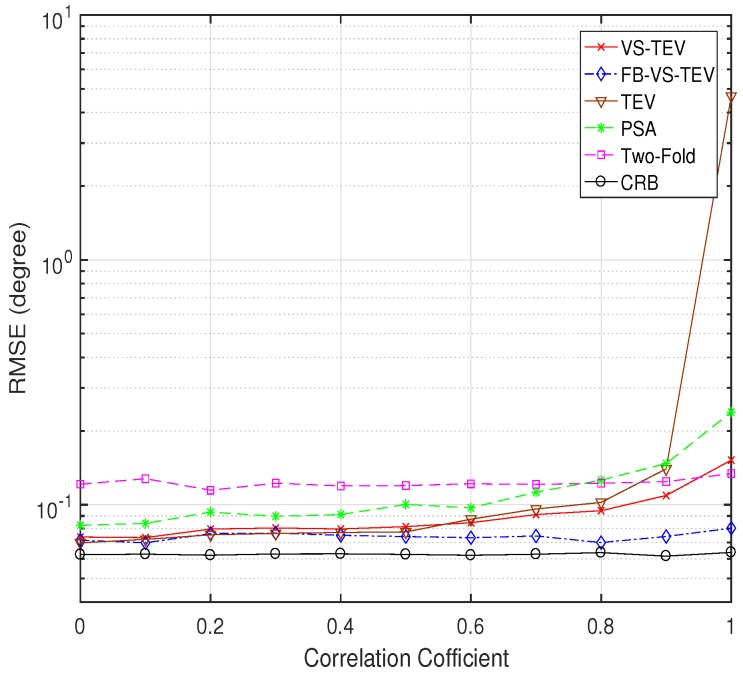
RMSE of elevation and azimuth versus correlation coefficient for two signals with T=10, SNR = 20 dB.

Next, we investigate the impact of the signal correlation on the DOA estimation performance. Note that the TEV method is also included in this simulation for comparison. The SNR is set as 20 dB, and the number of samples is fixed at T=10. The DOA and polarization parameters are the same as those in Figure 5. In Figure 12, we vary the signal correlation from 0 to 1, i.e, from uncorrelated signals to coherent signals. It can be seen that the FB-VS-TEV, VS-TEV, TEV methods have almost the same elevation and azimuth estimation performance when ρ≤0.5. When ρ≥0.4, the FB-VS-TEV method provides the best DOA estimation performance. Note that FB-VS-TEV and two-fold methods are relatively robust to the signal correlation. However, the TEV method becomes invalid when the signals are coherent.

### 5.3. The Case of Unideal Electromagnetic Vector-Sensor Array

Finally, we consider a complex DOA estimation scenario where both elements’ position errors and mutual coupling within each electromagnetic vector-sensor exist. The DOA and polarization information of two coherent signals are set as the same as those in Figure 5. Moreover, we set the elements position errors and mutual couplings as $\Delta =5\times 10^{-3}$ and δ=0.1, respectively.

**Figure 13:**
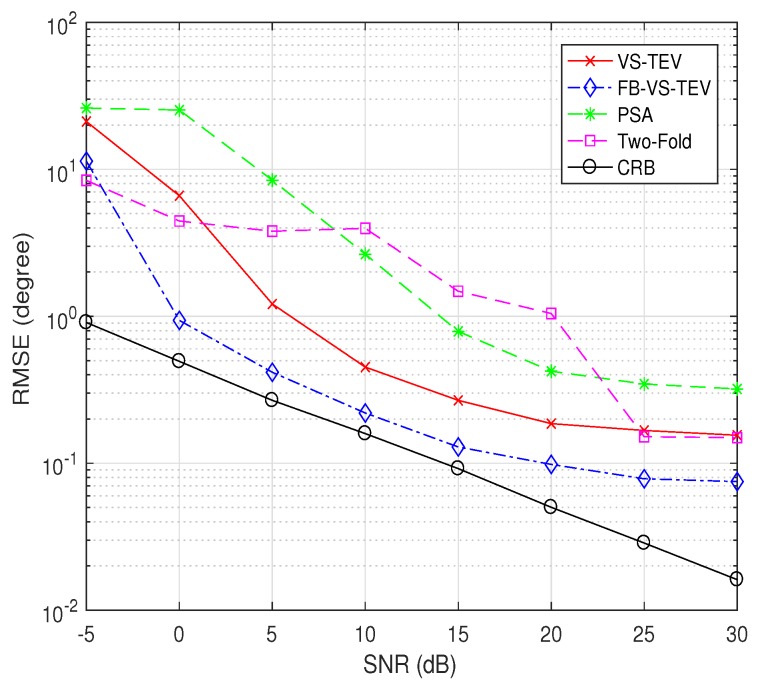
RMSE of elevation and azimuth versus SNR. The number of samples with T=10.

In Figure 13, RMSEs of the PSA, two-fold and proposed methods varying SNR are provided. We can observe that the FB-VS-TEV method has the best performance, and the VS-TEV method is superior to the other methods when SNR > 0 dB. The two-fold method has almost the same performance as the VS-TEV when SNR > 25 dB. As we can seen, the proposed two methods are more robust to errors than the other approaches.

**Figure 14:**
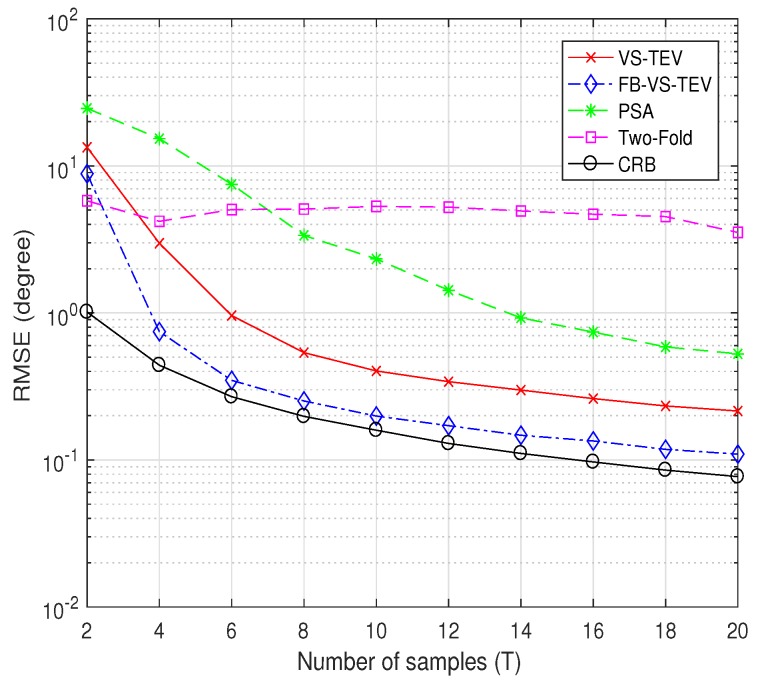
RMSE of elevation and azimuth versus the number of samples. The SNR is fixed at 10 dB.

In this experiment, we investigate the DOA estimation performance of the aforementioned four methods when the number of samples varies from 2 to 20, which is plotted in Figure 14. In this case, the SNR is fixed at 10 dB. It is easily seen that the FB-VS-TEV method has almost the lowest RMSE among the four methods. The VS-TEV method performs better than PSA and two-fold methods when T>4. In a word, the proposed methods are more robust to the elements’ position errors and mutual couplings.

## 6. Conclusions

In this paper, we address the DOA estimation problem using a polarized vector-sensor URA. A tensor-based eigenvector parameter estimation algorithm is derived. The proposed tensorial formulation models the polarized vector-sensor and the spatial phase factor of URA along different dimensions. It is shown that the proposed algorithms could handle coherent signals with polarized vector-sensor URA and demand modest computational burdens. Also, the automatic paring of DOAs and polarization parameters is achieved. Simulations are carried out to verify the effectiveness of the proposed algorithms.

## Figures and Tables

**Table 1:** Method for Direction-of-Arrival (DOA) Estimation with Polarized Vector-Sensor Array.

(i) Construct the *t*-th array measurement y(t) as in (6)
(ii) Fold y(t) to get Z(t) according to (12)
(iii) Build the corresponding tensor model as in (19)
(iv) Compute HOSVD of 𝓩 based on (23) to obtain the tensor-based signal subspace ${\hat{𝓤}}^{\left[s\right]}$
(v) Divide ${\hat{𝓤}}^{\left[s\right]}$ into *K* sub-tensors, then perform selection matrix to each dimension of sub-tensors based on (36)–(40)
(vi) Obtain estimates of elevation and azimuth through LS or SLS algorithm.

**Table 2:** Comparison of computation time.

	VS-TEV	FB-VS-TEV	PSA	Two-Fold
Average CPU time	0.113 s	0.123 s	0.033 s	18.421 s
